# *Podoplanin* hetero-insufficiency mice with inflammation in the jejunum demonstrates a good animal model of congenital protein-losing enteropathy

**DOI:** 10.1016/j.jphyss.2025.100031

**Published:** 2025-07-22

**Authors:** Toshio Ohhashi, Nagaharu Tsukiji, Moyuru Hayashi, Tomomi Watanabe-Asaka, Mieko Takasaka, Daisuke Maejima, Katsue Suzuki-Inoue, Yoshiko Kawai

**Affiliations:** a Department of Innovation of Medical and Health Sciences Research, Shinshu University School of Medicine, Matsumoto, Japan; b Department of Clinical and Laboratory Medicine, Faculty of Medicine, University of Yamanashi, Chuo, Yamanashi, Japan; c Division of Physiology, Faculty of Medicine, Tohoku Medical and Pharmaceutical University, Sendai, Japan

**Keywords:** Podoplanin, Protein-losing enteropathy, Jejunum, Permeant albumin

## Abstract

We have identified the Podoplanin expression in rat jejunal villi. We aimed to investigate the relationship between the Podoplanin expression in jejunal villi and the pathogenesis of congenital protein-losing enteropathy (PLE), using the Podoplanin heterozygous knock-out (*Pdpn*-het KO) mice and aspirin-induced inflammation of the jejunum. In the *Pdpn*-het KO mice the reticular and complexes distribution of Podoplanin was observed in the jejunal villi, resulting in be swollen of lamina propria. To confirm the abnormal distribution of small lymph vessels in the jejunal villi, the LYVE-1 immunohistochemical expression was investigated. The expression of Podoplanin and LYVE-1 in the jejunum of the *Pdpn*-het KO mice exhibited a distinct pattern. The intravenous administration of Evans blue dye appeared quickly into the mesenteric lymph vessels and lymph nodes in the wild-type but not observed in *Pdpn*-het KO mice. In the *Pdpn*-het KO mice, aspirin-induced jejunal inflammation produced a significant leakage of the intravenous administration of FITC-albumin into the jejunal lumen. The hypoalbuminemia in the blood and the marked distribution of FITC-albumin in the jejunal villi were also observed in the mice. In conclusion, we proposed that *Pdpn*-het KO mice with jejunal inflammation demonstrates a good animal model of the congenital PLE.

## Highlights

• Podoplanin hetero-insufficiency mice with jejunal inflammation demonstrates the similar symptom in the patients of congenital PLE.
• Based on the findings, podoplanin hetero-insufficiency mice with jejunal inflammation demonstrates a good animal model of the PLE.

## Graphical Abstract

**Figure:**
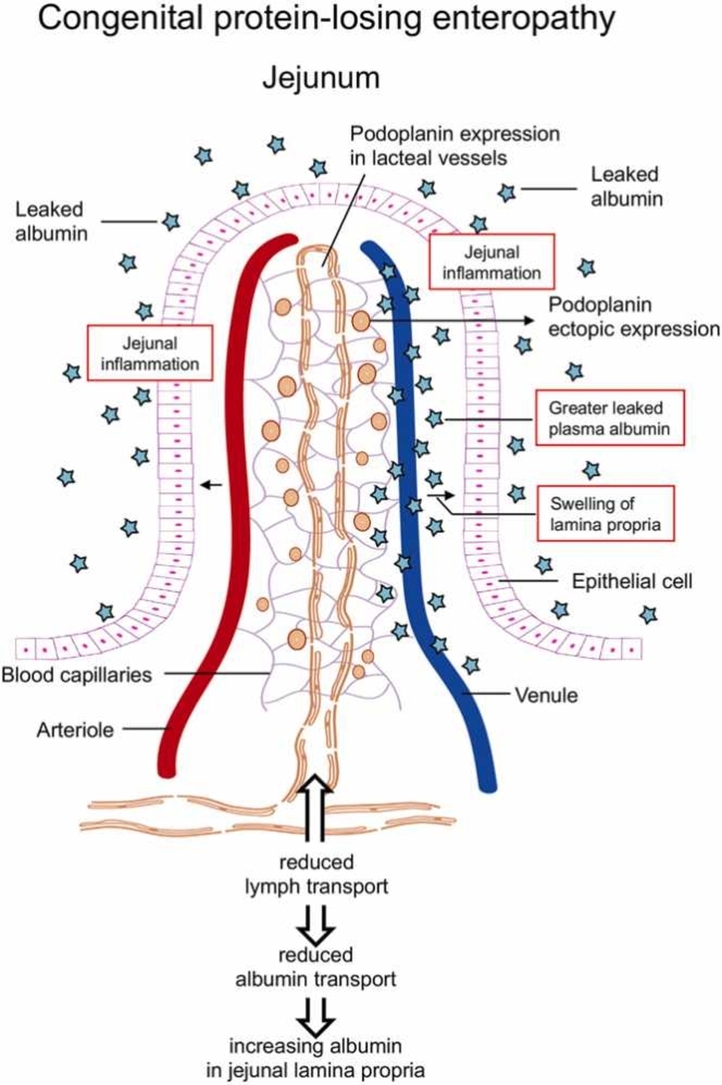
Graphical abstract of the conclusion in the present study. In the *Pdpn*-het KO mice, the aspirin-induced jejunal inflammation resulted in a significant leakage of the FITC-albumin into the jejunal lumen, resulted in the induction of marked hypoalbuminemia. In addition, the lymphangiectasia was confirmed in the lamina propria of jejunal villi, which disturbed mesenteric lymph flow and stored permeant albumin in jejunal villi. Thus, the *Pdpn*-het KO mice may be a good animal model of congenital PLE.

## Introduction

The jejunal microcirculation is characterized by specific properties including the movement of substantial quantities of albumin from the venular walls to surrounding tissues associated with its higher tissue osmotic pressure at the venular side [7], [8]. Jejunum-originated mesenteric lymph flow is also known to be larger than that in other organs [22]. Consistent with these properties, mesenteric collecting lymph vessels exhibit heart-like spontaneous contractions, facilitating the active transportation of large amounts of lymph fluid into the lacteal chyli [15], [16], [20]. Currently, we demonstrated that the higher permeability of albumin in the jejunal microcirculation may play pivotal roles in the transport of consumed water [13]. In addition, it has been demonstrated that portal venous blood flow-dependent shear stress stimulation induced the release of nitric oxide (NO) from venular endothelial cells of the rat jejunum, which plays physiologically crucial roles in the lymph formation of mesenteric lymphatic system [2]. We also demonstrated that water intake accelerated the release of serotonin from the enterochromaffin cells in rat jejunal villi and then transported through portal vein [11]. Therefore, serotonin circulating throughout the systemic circulation may be a crucial regulator in the mesenteric lymph formation.

Podoplanin was initially discovered in the lungs and on the surface of stromal cells in lymph nodes [4]. It has also been identified on the endothelium of lymph vessels [4], pneumocytes [18], and glomerular podocytes [4], [5], [6]. However, its presence in the subepithelial space of the jejunal villi had not yet been reported. Recently, we have identified the Podoplanin expression in the epithelial cells and the interepithelial space of rat jejunal villi [9]. We demonstrated that water drained through the epithelial cell layers in the jejunum led to a significant increase in Podoplanin transcripts and the protein expressions in the same region [9]. Nevertheless, the functional roles of Podoplanin in the interepithelial space of jejunal villi remain poorly understood. Given the physiological function of glomerular podocytes [4], the Podoplanin in the small intestine may be a key inhibitor in the movement of permeant plasma albumin presented in the interstitial tissues of jejunal villi into the jejunal lumen, the movement of which is found in patients of congenial protein-losing enteropathy (PLE, [17]).

The pathophysiology of congenital PLE is likely influenced by a combination of inflammatory, physiological and anatomic factors [3], [10], [19]. Thus, the structure of lymph capillaries in the jejunal villi shows a specific pattern which is suitable for absorption of water and nutrients [15], [16]. On the other hand, therapeutic approaches have generally directed towards addressing hypoproteinemia, the diminished integrity of intestinal mucosa, and the underlying altered hemodynamics [19]. A histological examination of the upper small intestine in the patients clarified that the lacteal cyst dilated and acute inflammation of crypts and lamina propria [17]. Mild lymphangiectasia was observed in all patients with PLE [17]. However, the complete pathogenesis of congenital PLE remains unclarified still now.

Based on these evidences, we have aimed to investigate whether or not the Podoplanin expression in the jejunum, abnormality of jejunal-originate lymph vessels and aspirin-induced jejunal inflammation in the Podoplanin heterozygous knock-out mice (*Pdpn*-het KO mice) may be a useful animal model of congenital PLE.

## Materials and methods

### In vivo mice experiments

All experimental protocols in the study were approved by the Institutional Animal Care and Use Committee of Shinshu University (No. 023017, 1 st April, 2019). Male and female C57/BL6JJmsSlc wild-type mice (10–15week old; Japan SLC, Sizuoka, Japan) and Podoplanin heterozygous knock-out (10–15week old, *Pdpn*-het KO) mice provided by Yamanashi University Faculty of Medicine [21] were fed a standard pellet diet (MF; Oriental Yeast, Tokyo) and water ad libitum. The animals were fasted overnight prior to the experiments. In the present experiments, male and female *Pdpn*-het KO mice were used. These mice were anaesthetized with 2.0 - 3.0 % isoflurane (Dainippon Sumitomo Pharma, Tokyo) and then placed on the operating table in a supine position for following experiments.

To induce the aspirin-mediated jejunal inflammation, 1 mg/kg aspirin (acetylsalicylic acid, A5376, Sigma-Aldrich: St Louis, [14]) suspended in ethyl-alcohol were administered daily intraperitoneal injection to wild-type and *Pdpn*-het KO mice for 4 or 5 days prior to the experiment.

### Observation of leaked FITC-albumin

To evaluate the FITC-labeled albumin (A9771, Sigma, St Louis) in the jejunum of the wild-type and *Pdpn*-het KO mice, the jejunum was rapidly isolated after tail venous injection of 0.2 mL FITC-albumin (1 mg/mL) and fixed with 4 % paraformaldehyde in physiological buffer solution (PBS, pH7.4) overnight. Subsequently, the fixed samples were then processed for paraffin-embedded and sectioned for further analyses.

### Quantitative analysis of leaked albumin

To evaluate the intravenous administration of FITC-labelled albumin (A9771, Sigma, St Louis) leaked into the jejunal lumen in the wild-type and *Pdpn*-het KO mice, after the aspirin induced jejunal inflammation, distilled water was infused into the intestinal cavity of the distal part of the stomach at a rate of 1 mL per minute. The polyethylene catheter was inserted at the middle portion of jejunum and the flow-through fluid was collected from the point. The fluid was collected every minute from 2 min before to 10 min after the intravenous administration of FITC-labelled albumin. The fluorescent intensity of each sample was measured using Power Scan (113441, Laboratory Ltd, Tokyo) for measuring the fluorescence intensity of FITC-labelled albumin in the collected sample to quantitatively evaluate the leakage of albumin into the jejunal lumen.

Evaluation for an intravenous administration of Evans blue dye into the mesenteric lymph vessels and lymph nodes, we investigated whether or not there was time difference in the appearance of leaked Evans blue dye following intravenous administration into the mesenteric lymph vessels and lymph nodes between the wild-type and *Pdpn*-het KO mice. To record the color change in mesenteric lymph vessels and lymph nodes after intravenous administration of 0.2 mL of 1 % Evans blue dye (056–01962, Wako, Osaka), the abdomen was opened by cutting along the midline. The mesenteric lymph vessels and lymph nodes were exposed by removing the surrounding adipose and connective tissues. The color changes within the lymph vessels and lymph nodes were recorded over time via video camera (Ricoh CX6, Tokyo).

### Histology

To evaluate the immunoreactivities of Podoplanin in the jejunum, lung, and kidney between wild- type and *Pdpn*-het KO mice, the aforementioned tissues were rapidly isolated and fixed with 4 % paraformaldehyde in PBS for overnight and then processed for 4 µm thick paraffin-embedded sections. These slices were rehydrated and incubated for overnight at 4 ºC with rat anti-mouse Podoplanin monoclonal antibody (015–24111; Fuji Film Wako, Osaka) diluted 1:500 after microwave treatment (500 W, 20 min). Then, slices were treated with anti-Rat IgG Alexa Flour 488 (A11006, Thermo Fisher Scientific, MA) diluted 1:500 for signal detection. The specificity of the antibodies was confirmed with the instructions in each catalog. Following three washes in PBS, the tissue slices were mounted with ProLong Gold antifade reagent and DAPI (P36935; Invitrogen, Carlsbad, CA) to counterstain the cell nuclei. Fluorescence was visualized using a Zeiss AxioImager M2 upright microscope. Separate images were taken and merged using ZEN 2.1 software.

To evaluate the co-localization of Podoplanin and LYVE-1 in the jejunum villi, the jejunum of wild- type and *Pdpn*-het KO mice were rapidly isolated and fixed with 4 % paraformaldehyde in PBS for overnight and then processed for 4 µm thick paraffin-embedded sections. These slices were rehydrated and incubated for overnight at 4 ºC with rat anti-mouse Podoplanin monoclonal antibody (015–24111; Fuji Film Wako, Osaka) diluted 1:500 after microwave treatment (500 W, 20 min) and followed by the treatment with anti-Rat IgG Alexa Flour 488 (Thermo Fisher Scientific, MA) diluted 1:200. After the immunohistostaining of the Podoplanin, Then, slices were treated with anti-LYVE-1(H-156) rabbit polyclonal antibody (sc-28190, Santa Cruz Biotec, USA) diluted 1:100 for 1.5 h and followed by anti-Rabbit IgG Alexa Flour 555 (A31572, Thermo Fisher Scientific, MA) diluted 1:200. After the staining, slices were mounted with ProLong Gold antifade reagent and DAPI (P36935; Invitrogen, Carlsbad, CA) to counterstain the cell nuclei and examined with a fluorescent microscope (BZ-9000; KEYENCE, Tokyo).

For the observation of the leaked FITC-albumin, anti-FITC antibody (#71–1900, Thermo Fisher Scientific, MA) diluted 1: 100 and anti-Rabbit IgG Alexa488 (A21441, Thermo Fisher Scientific, MA) diluted 1: 200 was used for the detection of FITC as the leaked albumin. The adjacent section was rehydrated and washed three times with PBS for Hematoxylin and eosin (HE) staining. Stained slices were examined with a fluorescent microscope (BZ-9000; KEYENCE, Tokyo).

### Western blotting analysis

To conduct Western blotting analyses of Podoplanin, the middle part of the jejunum from mice were lysed on ice using RIPA buffer (Boston BioProducts) supplemented with protease and phosphatase inhibitors (Roche). Following centrifugation, protein extracts were collected from the supernatant and were measured the total protein concentration with the Pierce BCA Protein Assay kit (Thermo Fisher Scientific). After equalizing the total protein mass, the protein solutions were mixed with NuPAGE LDS Sample Buffer (x 4) (Invitrogen) with 10 % β-mercaptoethanol and boiled at 95 °C for 10 min prior to loading onto 10 % SDS-PAGE and transferred to PVDF membranes. Precision Plus Protein Dual Color Standards (Bio-Rad) were used. Podoplanin were detected using rat anti- Podoplanin antibody (PMab1, 1:2000, Fujifilm Wako, 015–24111) as the primary antibody and an HRP conjugated anti-Rat IgG antibody (1:10,000, Novus biologicals, NBP1–73378) as the secondary antibody. ECLTM Prime (cytiva, RPN2232) was used for Chemiluminescent reaction and the resulting image was captured and analyzed using Amersham Imager 600 System (GE, USA).

### Measurement of serum albumin in the wild-type and *Pdpn*-het KO mice

To ascertain the reasonability of *Pdpn*-het KO mice as a selective animal model of congenital PLE to the correlation of human PLE, the concentration of albumin was measured in wild-type and *Pdpn*-het KO mice to confirm hypoalbuminemia as main symptom of human PLE [3], [10], [17], [19]. Blood samples from the both mice were obtained by cardiac puncture with a heparinized needle prior to and following the induction of aspirin-induced jejunal inflammation. The concentrations of serum albumin (mg/mL) were measured using the mouse albumin ELISA kit (Code No. 291–92301, FUJIFILM, Osaka).

### Drugs

All salts for PBS and PSS were obtained from Wako (Tokyo). Acetylsalicylic acid (aspirin) and heparin sulfate were obtained Sigma-Aldrich (St Louis).

### Statistical Analyses

All results are expressed as means ± standard error of the mean (SE). Statistical analysis was conducted with Student’s t test for unpaired observations after checking that the data conformed to at test distribution or one-way ANOVA followed by Bonferroni correction, as appropriate. P < 0.05 was considered statistically significant.

## Results

### Immunohistochemical reactivities of Podoplanin in the jejunum, lung, and kidney in the *Pdpn*-het KO and wild-type mice

**Fig. 1:**
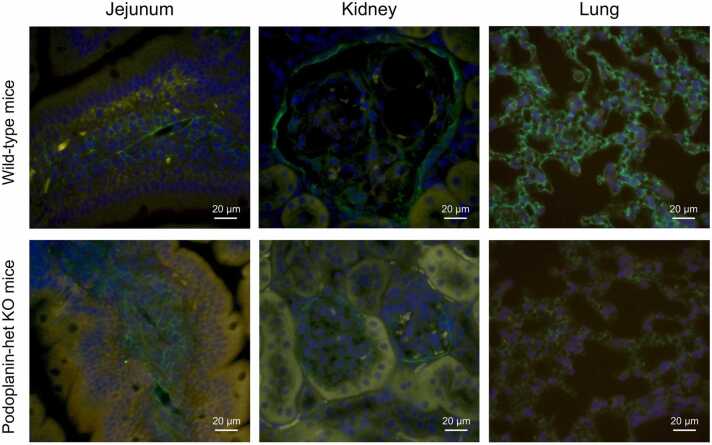
Representative photomicrographs of immunohistochemical images of Podoplanin with DAPI staining in the jejunum with high magnification (x 40, left panels), kidney (middle panels), and lung (right panels) in the wild -type (upper panels) and *Pdpn*-het KO (lower panels) mice. The green color is Podoplanin immunoreactivity. The blue color shows DAPI image.

To compare with the expression of Podoplanin in jejunum, kidney and lung in the *Pdpn*-het KO and wild-type mice, we investigated the immunohistochemical activates of jejunum, kidney and lung. Fig. 1 demonstrates representative photomicrographs of immunohistochemical images of Podoplanin in the jejunum, kidney, and lung in the wild-type (upper panels) and *Pdpn*-het KO mice (lower panels). The green-colored immunoreactivities of Podoplanin were clearly observed in the lamina propria of the jejunal villi in the both mice (Jejunum, left panels). The reticular patterns of Podoplanin were also confirmed in the jejunal villi in the both mice. In contrast, in the *Pdpn*-het KO mice the reticular distribution of Podoplanin seemed to be enlarged and complexes, resulting in be swollen of lamina propria of jejunal villi.

In contrast, no change in the immunoreactivities of Podoplanin of the glomerular podocytes was observed in wild-type and *Pdpn*-het KO mice. The immunoreactivity of Podoplanin in alveolar pneumocytes in the *Pdpn*-het KO mice were markedly suppressed compared with those in the wild-type mice.

### Western blotting for Podoplanin in the jejunal villi in the *Pdpn*-het KO and wild-type mice

**Fig. 2:**
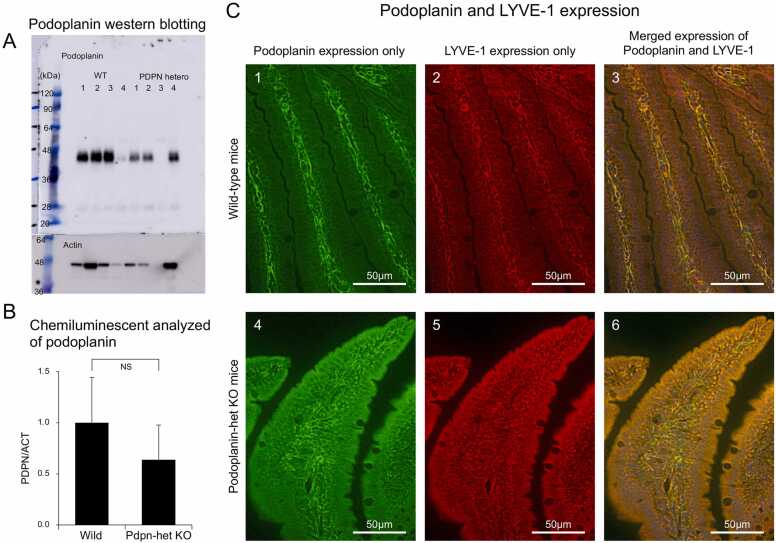
A: Representative data of Podoplanin Western blotting in the lamina propria of jejunal villi with the wild-type [1], [2], [3] and *Pdpn*-het KO [1], [2], [4] each three of mice. B: The chemiluminescent analyses of Podoplanin for the Western blotting in the wild-type and *Pdpn*-het KO mice. C 3&6: Representative immunoreactive expression of both Podoplanin (Green) and LYVE-1 (Red) in the jejunum of wild-type (upper) and *Pdpn*-het KO (lower) mice. C 1&4: Representative of immunoreactive expression of Podoplasnin only. C 2&5: Representative of immunoreactive expression of LYVE-1 only.

To confirm again the expression of Podoplanin in the jejunum, we conducted Western blotting analysis. Fig. 2A shows representative data of Podoplanin western blotting in the lamina propria of jejunal villi in wild-type and *Pdpn*-het KO mice. Podoplanin expression in the lamina propria of the jejunal villi in the wild-type mice seemed to be greater than those in the *Pdpn*-het KO.

As shown in Fig. 2B, the T value of Podoplanin in the Western blotting analysis in the wild-type mice was larger, but not significant, in compared with that in the *Pdpn*-het KO mice (1.00 ^+^ 0.44 in wild-type mice vs 0.63 + 0.34 in *Pdpn*-het KO, each 3 animals, NS n = 3).

### Distribution of LYVE-1 in the jejunum villi in the *Pdpn*-het KO and wild-type mice

To confirm whether or not there are the abnormalities in the distribution of small lymph vessels in the jejunal villi, the immunohistochemical expression of LYVE-1 and Podoplanin were simultaneously investigated in the jejunal villi. Fig. 2C (3 & 6) demonstrate the representative of immunoreactive expression s of merged LYVE-1 (red color) and Podoplanin (green color) in the jejunal villi of the *Pdpn*-het KO (lower) and wild-type mice (upper). Fig. 2C (1 & 4) and (2 &5) show the each Podoplanin and LYVE-1 expression only in the same section of the both mice.

The jejunal villi in the *Pdpn*-het KO seemed to be larger than wild-type, we measured the width of lamina propria at the midpoint along the length of jejunal villi in the wild-type and *Pdpn*-het KO mice (57.1 + 7.8 µm in wild-type and 109.0 + 16.8 µm in *Pdpn*-het KO, 3 mice, each 2 sections, p < 0.01 n = 6, respectively). This is result indicated that the jejunal villi in the Pdpn-het KO is swollen.

### Evaluation of leaked FITC-albumin in the jejunal lumen in the *Pdpn*-het KO and wild-type mice with and without aspirin-induced jejunal inflammation

**Fig. 3:**
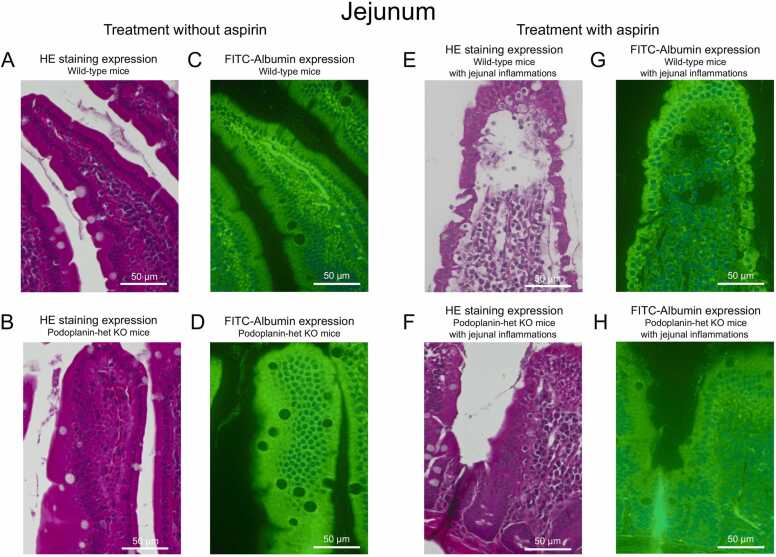
A B: Representative photomicrographs of HE-stained histological images of the jejunal villi wild-type(upper) and *Pdpn*-het KO (lower) mice without the treatment of aspirin. 3 C, D: Representative photomicrographs of fluorescent expression of FITC-albumin using FITC antibody in the jejunum of wild-type (upper) and *Pdpn*-het KO (lower) mice without the treatment of aspirin. 3 E, F: HE-stained histological image of the jejunal villi in the *Pdpn*-het (lower) and the wild-type (upper) mice with aspirin-induced jejunal inflammation, respectively. 3 G, H: Fluorescent image using FITC antibody in the *Pdpn*-het (lower) and the wild-type (upper) mice with the jejunal inflammation, respectively.

Next, we investigated whether or not the Podoplanin expression produced the modification of distribution and concentration of intravenous administration of FITC-albumin in the jejunal villi of *Pdpn*-het KO and wild-type mice. Fig. 3A and B shows the HE-staining of jejunal preparations as wild-type mice (upper) and *Pdpn*-het KO mice (lower) without the jejunal inflammation.

The fluorescent expression of FITC-albumin in the jejunum of *Pdpn*-het KO mice were markedly increased in comparison with wild-type mice by the antibody staining to FITC (Fig. 3C & 3D). The fluorescent activities of FITC-albumin in the interepithelial space and lamina propria of jejunal villi in the *Pdpn*-het KO mice were observed more markedly (Fig. 3D).

We additionally investigated whether or not the jejunal inflammation with aspirin exacerbated.

albumin leakage in the jejunal villi in the *Pdpn*-het KO and wild-type mice. Fig. 3E–H demonstrate representative HE-stained histological (left panels) and fluorescent images (right panels) of the jejunal villi in the wild-type (upper) and *Pdpn*-het KO mice (lower) with the jejunal inflammation, respectively. The HE-staining images were obtained with the consecutive serial sections of the FITC immunoreactivity preparation of Podoplanin. As shown in Fig. 3F, the small isolation of epithelial cell layer and disappearance of several cells in the lamina propria were observed in the jejunum of the *Pdpn*-het KO mice. In addition, the leaked FITC-albumin was found on the surface of interepithelial cell layer in the jejunum of the *Pdpn*-het KO mice (Fig. 3H).

Fig. 3E shows representative HE staining images of the jejunal villi in the wild-type mice with the jejunal inflammation. In the similar to the *Pdpn*-het KO mice, the disappearance of cells in the lamina propria of jejunal villi were markedly observed. In contrast, in the fluorescent image of leaked FITC-albumin was not clearly observed in the wild-type mice (Fig. 3G).

### Evaluation for movement of an intravenous administration of Evans blue dye into the mesenteric lymph vessels and lymph nodes in the *Pdpn*-het KO and wild-type mice

To clarify the mechanisms underlying the higher concentration of albumin in the jejunal villi of *Pdpn*-het KO mice, we measured a time-course of the movement of blue-colored albumin to appear into the mesenteric lymph vessels and lymph nodes in the both mice. Because the intravenously administered Evans blue dye was known to transport rapidly combined with permeant plasma albumin into the interstitial tissue of jejunum [13].

**Fig. 4:**
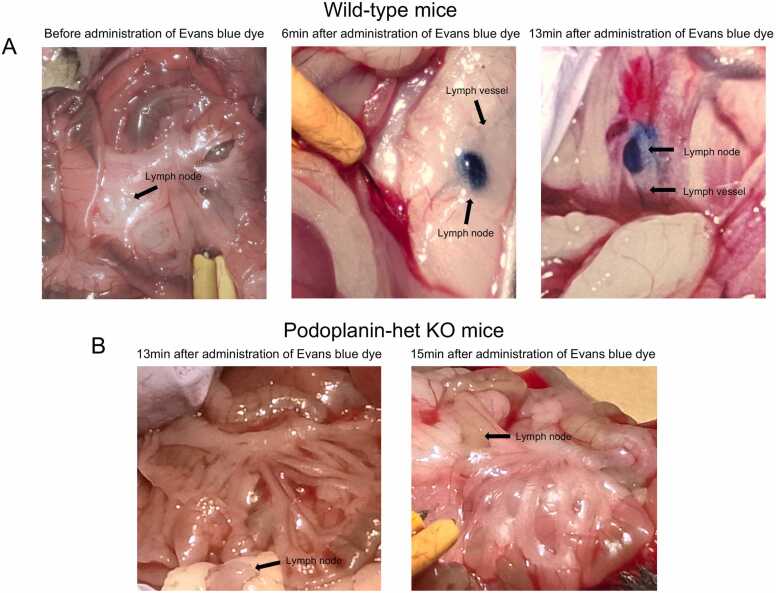
A: Representative photomicrographs of Evans blue dye in the mesenteric lymph vessel and lymph node of wild-type mice (upper panels) before (left panel), at 6 min (middle panel) and 13 min (right panel) after intravenous administration of Evans blue dye. 4B: Representative photomicrographs of the mesentery of *Pdpn*-het KO mice (lower panels) at 13 min (right panel) and 15 min (left panel) after intravenous administration of Evans blue dye. No blue-colored mesenteric lymph vessel and lymph node was observed.

Fig. 4 demonstrates the representative photomicrographs of Evans blue dye appeared into the mesenteric lymph vessels and lymph nodes in the wild-type (Fig. 4A) and *Pdpn*-het KO mice (Fig. 4B). As shown in Fig. 4A left panel, the lymph vessels and lymph nodes were not stained in blue color before the administration. In contrast, the blue-colored lymph vessels and large lymph node were observed markedly in wild-type mice approximately 6 min after intravenous administration of Evans blue dye (Fig. 4A middle panel). The Evans blue dye into the lymph node became darker and dense approximately 13 min after the administration (Fig. 4A right panel). This phenomenon was confirmed in all 5 wild-type mice.

In contrast, no identified blue-colored lymph vessels and lymph nodes were observed until 13 and 15 min after the administration of Evans blue dye in all 5 *Pdpn*-het KO mice (Fig. 4B, right and left panels). The no blue color lymph vessel and lymph node was found in all 5 *Pdpn*-het KO mice.

### Intravenous administration of FITC-albumin-dependent fluorescent intensity of distilled water perfused through jejunal lumen

**Fig. 5:**
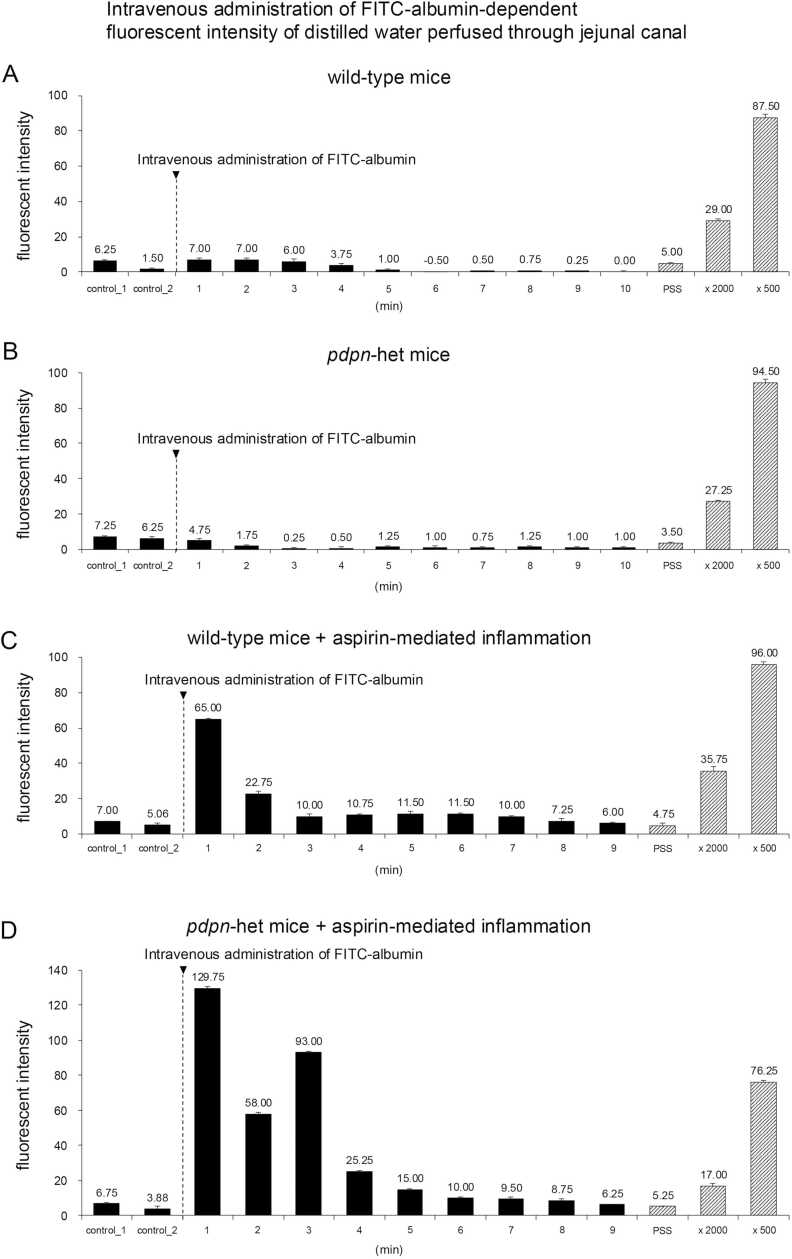
Representative recordings of time-dependent changes in fluorescent intensity of collected the fluid perfused through jejunal lumen before (control 1 and 2) and 1 min each after intravenous administration of FITC-albumin in the wild-type (5 A: upper panel) and *Pdpn*-het KO mice (5 C lower panel) without aspirin-induced jejunal inflammation. The arrow head is the starting point of intravenous administration of FITC-albumin (0.15 mL). The distilled water was injected into the stomach of mice at each time. The PSS is the fluorescent intensity of physiological saline solution. The x 2000 and x 500 show the fluorescent intensity of each solution diluted FITC-albumin with distilled water. 5B and 5D show the same time-dependent changes in the fluorescent intensity in the wild-type and *Pedon*-het mice with the jejunal inflammation, respectively.

The fluorescent intensity in perfused distilled water through the jejunal lumen was measured each 1 min over 2 min before and 10 min after the intravenous administration of FITC-labeled albumin in the wild-type and *Pdpn*-het KO mice without and with the jejunal inflammation. Fig. 5 shows the mean value (each 4 measurements) of the fluorescent intensity of distilled water perfused through the jejunum lumen at each time. The slight fluorescent activities in the fluid were confirmed in both mice before the administration (Fig. 5 control 1 and 2). The mean values of the fluorescent intensities of the perfused fluid in the control were similar to be the fluorescent activity with PSS (6.0 + 1.1 in *Pdpn*-het KO mice vs 5.0 + 1.8 in wild-type; NS n = 8, each value vs 4.0 + 1.6 in PSS, NS n = 8). In addition, no significant difference of total fluorescent intensities during 10 min after the administration of FITC-albumin was confirmed between wild-type and *Pdpn*-het mice without the jejunal inflammation (10.7 + 0.3 in wild-type vs 10.2 + 1.1 in *Pdpn*-het KO, NS n = 8).

In contrast, the pretreatment with aspirin produced significant increases in the total fluorescent intensities of perfused water during 10 min after the administration of FITC-albumin in the wild-type and *Pdpn*-het KO mice (Figs. 5A, 5C: 10.7 + 0.3 vs 13.3 + 1.6 wild-type, p < 0.05 n = 8; Figs. 5B, 5D: 10.2 + 1.1 vs 41.9 +1.3 in *Pdpn*-het KO, p < 0.01 n = 8). In addition, in the *Pdpn*-het KO mice the total 10 min fluorescent intensities of leaked FITC-albumin into the jejunal lumen were significantly greater than that in the wild-type mice (Figs. 5C, 5D: wild- type mice 13.3 + 1.6 vs *Pdpn*-het KO mice 41.9 + 1.3, p < 0.01 n = 8).

### Evaluation of the concentration of serum albumin in the *Pdpn*-het KO and wild-type mice with and without aspirin-induced jejunal inflammation

To evaluate the changes in the concentration of serum albumin with and without jejunal inflammation, we measured the concentration of albumin in blood of *Pdpn*-het KO and wild-type mice with and without jejunal inflammation.

**Fig. 6:**
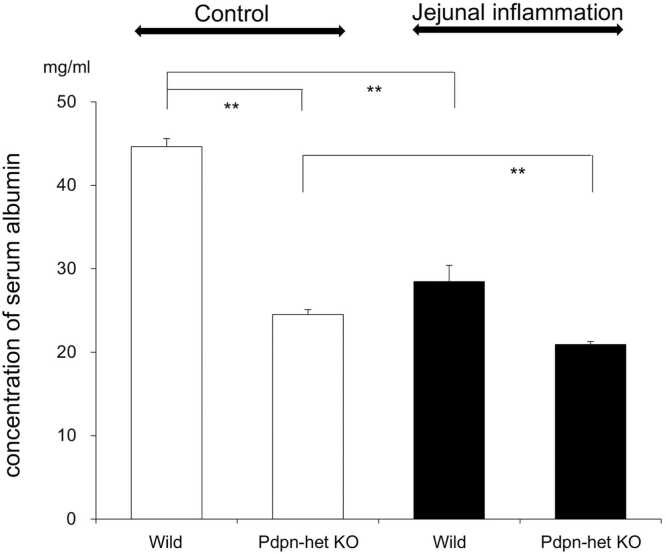
The summarized data (3 mice, n = 6) of concentrations of serum albumin in the *Pdpn*-het KO and wild- type mice with (black columns) and without (white columns) aspirin-induced jejunal inflammation. ** p < 0.01.

Fig. 6 shows the summarized data of the concentration of albumin in the *Pdpn*-het KO and wild-type mice with (Jejunal inflammation, black columns) and without (Control, white columns) aspirin-induced jejunal inflammation. In physiological conditions (Control in Fig. 6), a marked hypoalbuminemia was observed in the *Pdpn*-het KO mice compared with that in the wild-type (24.5 + 0.6 mg/mL in *Pdpn*-het mice vs 44.6 + 1.0 mg/mL in wild-type, 3 mice, each 2 samples p < 0.01 n = 6). The presence of jejunal inflammation accelerated more marked hypoalbuminemia in the *Pdpn*-het mice (24.5 + 0.6 mg/mL without inflammation vs 20.9 + 0.3, mg/mL, 3 mice, each 2 samples p < 0.01 n = 6). Similar acceleration of hypoalbuminemia was observed in the wild-type mice with jejunal inflammation (44.6 + 1.0 mg/mL without inflammation vs 28.5 + 1.1 mg/mL with inflammation,

3 mice, each 2 samples p < 0.01 n = 6).

## Discussion

### New findings of the jejunum in the *Pdpn*-het KO mice

In the present experiments, based on the evidence that Podoplanin expression has confirmed in the interepithelial cell space of rat jejunal villi [9], and that the physiological function of glomerular podocytes has been well known [4], we aimed to clarify the pathogenesis of congenital PLE using the *Pdpn*-het KO mice [22]. The *Pdpn*-het KO mice exhibited a significant reduction in Podoplanin expression in pulmonary pneumocytes while no or minimal change in kidney podocytes (Fig. 1A). In addition, the immunoreactivity of Podoplanin in the jejunal villi was also confirmed in the lamina propria of jejunal villi in the *Pdpn*-het KO mice.

**Fig. 7:**
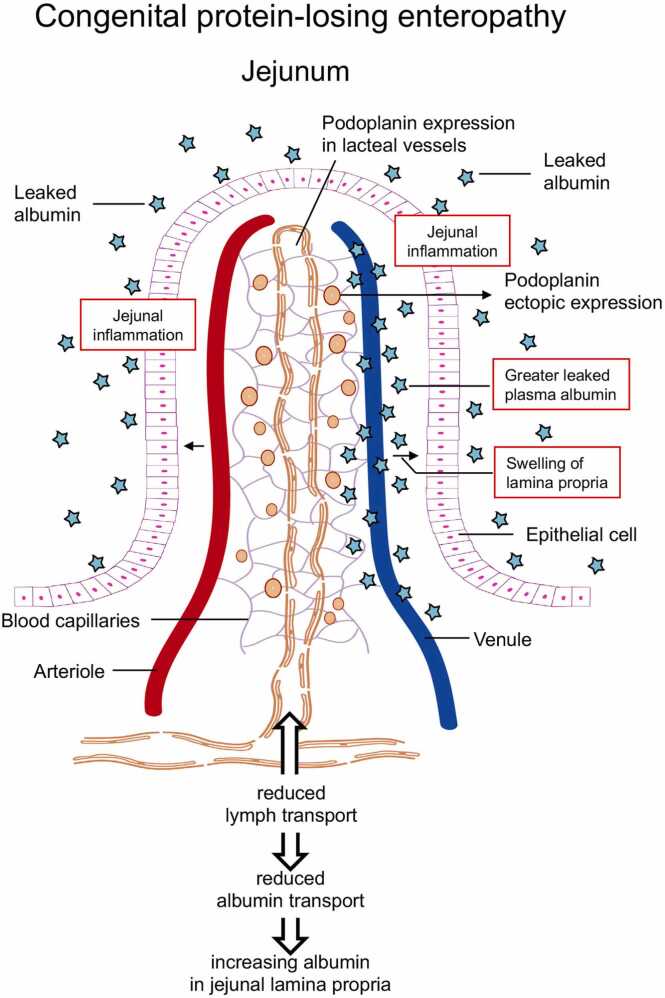
Graphical abstract of the conclusion in the present study. In the *Pdpn*-het KO mice, the aspirin-induced jejunal inflammation resulted in a significant leakage of the FITC-albumin into the jejunal lumen, resulted in the induction of marked hypoalbuminemia. In addition, the lymphangiectasia was confirmed in the lamina propria of jejunal villi, which disturbed mesenteric lymph flow and stored permeant albumin in jejunal villi. Thus, the *Pdpn*-het KO mice may be a good animal model of congenital PLE.

In *Pdpn*-het KO mice, western blotting revealed a significant decrease in Podoplanin expression in the jejunal villi. This decrease in Podoplanin expression was concomitant with remarkable swelling of the lamina propria of the lacteal vessels, as well as with leakage of plasma albumin from the jejunal villi in the aspirin-induced inflammation. These finding suggests a potential role for decreased expression of Podoplanin in the congenital protein-losing enteropathy (Fig. 7 as a graphical abstract).

### A significant increase in the distribution of permeant albumin in the jejunum in the *Pdpn*-het mice

In physiological conditions, there exists specific properties of jejunal micro- and lymph-circulation in the jejunum. Thus, the movement of large amounts of permeant plasma albumin from the venular walls to interstitial tissues in the jejunal villi [7], [8], resulting in larger lymph formation [22] and a lot of lymph flow in mesenteric lymph vessels [15], [16]. Currently, we demonstrated that the jejunal- originated lymph included large amounts of albumin has transported through chylocyst and thoracic duct into venous blood in human beings and rabbits [1], [12].

On the other hand, the Podoplanin expression in the epithelial cells and the interepithelial space of rat jejunal villi is markedly observed. We also demonstrated that the absorbed water drained through the epithelial cell layers in the jejunum led to a significant increase in the Podoplanin transcripts and the protein expressions in the jejunal villi, which are accelerated with water intake [9]. Given the physiological function of glomerular podocytes [4], the Podoplanin in the small intestine may be a key inhibitor in the movement of permeant plasma albumin presented in the interstitial tissues of jejunal villi into the lumen of small intestine.

In the present experiments, the large amounts of permeant albumin were accumulated in the lamina propria of the jejunal villi in the *Pdpn*-het KO mice (Fig. 3). The great deposition of permeant albumin in the jejunal villi may produce an increase of oncotic pressure and swelling in the tissues. The swelling-mediated physical force may contribute to stagnate or reduce the lymph flow from lacteal vessels in jejunal villi to mesenteric lymph vessels and lymph nodes. In fact, we confirmed in the *Pdpn*-het KO mice that the lymph transport included permeant albumin from the lacteal vessels to mesenteric lymph vessels and lymph nodes was markedly reduced (Fig. 4). These findings suggest that typical hypoproteinemia and low oncotic pressure in blood may be appeared in the *Pdpn*-het KO mice. In fact, a marked hypoalbuminemia was confirmed in the *Pdpn*-het KO mice, which was accelerated by the jejunal inflammation (Fig. 6). The hypothesis is completely agreed with clinical symptom in congenital PLE. Thus, in the PLE patients the enteric protein loss is increased and vascular oncotic pressure is decreased [3], [10], [19]. Therapeutic approaches have generally targeted hypoproteinemia [19]. Thus, the *Pdpn*-het KO mice may be a suitable animal model in the correlation with human PLE.

However, it may be possible concept that the damage of liver in the *Pdpn*-het KO mice contribute, in part, to produce the marked hypoalbuminemia in the mice. We will be needed in the future to evaluate this possible mechanism.

### A significant leakage of intravenous administration of FITC-albumin into the jejunal lumen in the *Pdpn*-het KO mice with aspirin-mediated jejunal inflammation

To confirm the hypothesis that the *Pdpn*-het KO mouse with inflammation is a good model for congenital PLE animal model, we investigated whether or not the intravenous administration of FITC-albumin leaks into the jejunal lumen. However, no or little fluorescent activity was observed in the fluid perfused through jejunal lumen in wild- type and *Pdpn*-het KO mice without jejunal inflammation (Fig. 5).

In contrast, the pretreatment with aspirin-mediated jejunal inflammation caused a significant leakage of FITC-albumin detected into the water through jejunal lumen in wild-type and *Pdpn*-het KO mice (Fig. 5). In agreement with the evidence, the histological analysis of the jejunal villi in the *Pdpn*-het KO mice treated with aspirin demonstrates marked jejunal inflammation with leaked the FITC-albumin on the surface of epithelial layer in the jejunal villi (Fig. 3). Thus, in the *Pdpn*-het KO mice, aspirin-induced jejunal inflammation resulted in a significant leakage of the FITC-albumin into the jejunal lumen. In addition, the *Pdpn*-het KO mice showed marked hypoalbuminemia which was similar to the typical symptom and pathogenesis in congenital PLE patients. It can be emphasized that the *Pdpn*-het KO mouse with inflammation is useful pathological models for elucidation of congenital PLE.

In conclusion, we proposed that Podoplanin het-KO mice in conjunction with jejunal inflammation demonstrates a good animal model of the congenital PLE. In fact, the patients with congenital PLE are known to appear clinical symptom compatible with the present findings such as hypoalbuminemia, and the disturbed integrity of small lymph vessels and lymphangiectasia in jejunal villi.

## CRediT authorship contribution statement

**Yoshiko Kawai:** Writing – review & editing, Visualization, Validation, Supervision, Project administration, Methodology, Investigation, Formal analysis, Data curation, Conceptualization. **Katsue Suzuki-Inoue:** Writing – review & editing, Visualization, Validation, Supervision, Resources, Project administration, Methodology, Investigation, Formal analysis, Data curation, Conceptualization. **Daisuke Maejima:** Writing – review & editing, Visualization, Validation, Supervision, Project administration, Methodology, Investigation, Formal analysis, Data curation, Conceptualization. **Mieko Takasaka:** Writing – review & editing, Visualization, Validation, Project administration, Methodology, Investigation, Formal analysis, Data curation, Conceptualization. **Moyuru Hayashi:** Writing – review & editing, Visualization, Validation, Supervision, Project administration, Methodology, Investigation, Formal analysis, Data curation, Conceptualization. **Tomomi Watanabe-Asaka:** Writing – review & editing, Visualization, Validation, Project administration, Methodology, Investigation, Formal analysis, Data curation, Conceptualization. **Nagaharu Tsukiji:** Writing – review & editing, Visualization, Validation, Resources, Project administration, Methodology, Investigation, Formal analysis, Data curation, Conceptualization. **Toshio Ohhashi:** Writing – original draft, Visualization, Validation, Supervision, Project administration, Methodology, Investigation, Funding acquisition, Formal analysis, Data curation, Conceptualization.

## Author contributions

T.O., T. W.-A. wrote the manuscript, designed the experiments, and analyzed the data. M.T., N.T., M.H., T.W-A., D.M., K.S-I., Y.K. designed the experiments, analyzed the data, and revised manuscript. M.T., T. W-A, M.H., D.M. and T.O. performed the experiments, and analyzed data.

## Consent for publication

All authors approved the final version of the manuscript and accepted for the publication of this manuscript.

## ARRIVE guideline

The study is reported in accordance with ARRIVE guideline.,

## Ethical approval

This study and all experimental protocols were approved by the Institutional Animal Care and Use Committee in Shinshu University (No.023017, 1st April, 2019).

## Funding

The Department of Innovation of Medical and Health Sciences Research at Shinshu University School of Medicine has been established and supported financially by the donation of BOURBON, Co., Ltd, Kashiwazaki, Niigata, Japan. The authors declare that this study received funding from BOURBON Co. Ltd. The funder was not involved in the study design, collection, analysis, interpretation of data, the writing of this article or the decision to submit it for publication.

## Declaration of Competing Interest

No conflicts of interest, financial or otherwise, are declared by the authors.

## Data Availability

All relevant data are available from the corresponding author, Toshio Ohhashi, on request.

## References

[1] Ajima K., Kawai Y., Maejima D., Suzuki S., Yano S., Hayashi M. (2018). Lymph drainage from the chylocyst -induced hemodilution in an in vivo rabbit study. Lymphatic Research and Biology.

[2] Amari K., Kajihara R., Arai N., Hayashi M., Watanabe-Asaka T., Kaidoh M. (2022). Portal blood flow-dependent NO-mediated lymph formation in rat jejunum. Pfügers Archiv - European Journal of Physiology.

[3] Braamskamp M.J.A.M., Dolman K.M., Tabbers M.M. (2010). Clinical practice. Protein-losing enteropathy in children. European Journal of Pediatrics.

[4] Breiteneder-Geleff S., Soleiman A., Kowalski H., Horvat R., Amann G., Kriehuber E. (1999). Angiosarcomas express mixed endothelial phenotypes of blood and lymphatic capillaries: podoplanin as a specific marker for lymphatic endothelium. The American Journal of Pathology.

[5] Breiteneder-Geleff S., Matsui K., Soleiman A., Meraner P., Poczewski H., Kalt R. (1997). Podoplanin, novel 43-kd membrane protein of glomerular epithelial cells, is down- regulated in puromycin nephrosis. The American Journal of Pathology.

[6] Farr A.G., Berry M.L., Kim A., Nelson A.J., Welch M.P., Aruffo A. (1992). Characterization and cloning of a novel glycoprotein expressed stromal cells in T-dependent areas of peripheral lymphoid tissues. Journal of Experimental Medicine.

[7] Granger D.N., Barrowman J.K. (1983). Microcirculation of the alimentary tract. I. Physiology of transcapillary fluid and solute exchange. Gastroenterology.

[8] Guyton A.C., Taylor A., Grange H.J. (1975).

[9] Hayashi M., Watanabe-Asaka T., Nagashio S., Kajihara R., Amari K., Yokoyama Y. (2021). Water intake accelerates ATP release from myofibroblast cells in rats: ATP-mediated podoplanin-dependent control for physiological function and immunity. American Journal of Physiology-Gastrointestinal and Liver Physiology.

[10] Johnson J.N., Driscoll D.J., O’Leary P.W. (2012). Protein-losing enteropathy and the Fontan operation. Nutrition in Clinical Practice.

[11] Kajihara R., Amari K., Arai N., Nagashio S., Hayashi M., Watanabe-Asaka T. (2021). Water intake releases serotonin from enterochromaffin Cells in rat jejunal villi. Pfügers Archiv - European Journal of Physiology.

[12] Kawai Y., Ajima K., Nagai T., Yokoyama Y., Kaidoh M., Seto E. (2015). Abdominal respiration induces hemodilution and reduction in ADH concentration of blood. Lymphatic Research and Biology.

[13] Nagashio S., Ajima K., Maejima D., Sanjo H., Kajihara R., Hayashi M. (2019). Water intake increases mesenteric lymph flow and the total flux of albumin, long-chain fatty acids, and IL-22 in rats: new concept of absorption in jejunum. American Journal of Physiology-Gastrointestinal and Liver Physiology.

[14] Nonoyama K., Nakagawa K., Amagase K., Takeuchi K., Nakamura M., Okabe S. (2010). New method of inducing lesions in rats by intraduodenal administration of aspirin. Journal of Gastroenterology and Hepatology.

[15] Ohhashi T., Mizuno R., Ikomi F., Kawai Y. (2005). Current topics of physiology and pharmacology in the lymphatic system. Pharmacology & Therapeutics.

[16] Ohhashi T., Kawai Y. (2015). Proposed new lymphology combined with lymphatic physiology, innate immunology, and oncology. The Journal of Physiological Sciences.

[17] Pelletier V.A., Galeano N., Brochu P., Morin C.L., Weber A.M., Roy C.C. (1986). Secretory diarrhea with protein-losing enteropathy, enterocolitis cystica superficialis, intestinal lymphangiectasia, and congenital hepatic fibrosis: a new syndrome. The Journal of Pediatrics.

[18] Ramirez M.I., Millien G., Hinds A., Cao Y.X., Seldin D.C., Williams M.C. (2023). T1 alpha, a lung type I cell differentiation gene, is required for normal lung cell proliferation and alveolus formation at birth. Developmental Biology.

[19] Rychik J., Spray T.L. (2002). Strategies to treat protein-losing enteropathy. Seminars in Thoracic and Cardiovascular Surgery: Pediatric Cardiac Surgery Annual.

[20] Simmonds W.J. (1955). Some observations on the increase in thoracic duct lymph flow during intestinal absorption of fat in un-anaesthetized rats. Australian Journal of Experimental Biology and Medical Science.

[21] Tsukiji N., Inoue O., Morimoto M., Tatsumi N., Nagatomo H., Ueta K. (2018). Platelets play an essential role in murine lung development through Clec-2/podoplanin interaction. Blood.

[22] Yoffey J.M., Courtice F.C. (1970).

